# What Are “Lingzhi Wang” or “Zhu Lingzhi”? Notes on *Ganoderma* (Ganodermataceae, Polyporales) Species Characterized by Diminutive Pilei and Gracile Stipes from Hainan Island, Tropical China

**DOI:** 10.3390/jof11030237

**Published:** 2025-03-20

**Authors:** Run Tian, Hua-Zhi Qin, Qing Zhong, Zhi-Qun Liang, Xue-Yan Zhang, Xiao-Dong Mu, Lin Xu, Ting-Chi Wen, Xiang-Dong Chen, Nian-Kai Zeng

**Affiliations:** 1 Ministry of Education Key Laboratory for Ecology of Tropical Islands, Key Laboratory of Tropical Animal and Plant Ecology of Hainan Province, College of Life Sciences, Hainan Normal University, Haikou 571158, China; tr121344@163.com (R.T.); huazhiqin2022@163.com (H.-Z.Q.); 202208071339@hainnu.edu.cn (Q.Z.); zhangxueyan_caas@126.com (X.-Y.Z.); 2 Institute of Medicinal Plant Development, Chinese Academy of Medical Sciences & Peking Union Medical College, Beijing 100193, China; 3 Qinghai Provincial Key Laboratory of Tibetan Medicine Pharmacology and Safety Evaluation, Northwest Institute of Plateau Biology, Chinese Academy of Sciences, Xining 810008, China; 4 University of Chinese Academy of Sciences, 19(A) Yuquan Road, Beijing 100049, China; 5 School of Chemistry and Chemical Engineering, Hainan University, Haikou 570228, China; lizhqu1980@126.com; 6 Hainan Research Academy of Environmental Sciences, Haikou 571126, China; hnmuxd@hainan.gov.cn; 7 Environmental and Plant Protection Institute, Chinese Academy of Tropical Agricultural Sciences, Haikou 571101, China; xulin@catas.cn; 8 State Key Laboratory of Green Pesticide, Key Laboratory of Green Pesticide and Agricultural Bioengineering, Ministry of Education, Guizhou University, Guiyang 550025, China; tingchiwen@yahoo.com; 9 Science and Technology Backyard for Edible and Medicinal Fungi in Baisha, Hainan Province, Baisha 572800, China; 10 Science and Technology Backyard for Lingzhi in Baisha, Hainan Province, Baisha 572800, China

**Keywords:** medicinal fungi, molecular phylogeny, morphology, new taxa, taxonomy

## Abstract

Species of *Ganoderma* (Ganodermataceae, Polyporales) have been extensively utilized in traditional Chinese medicine for over two millennia, owing to their remarkable medicinal properties and diverse chemical constituents. Hainan Island, located in tropical China, harbors a rich diversity of *Ganoderma* species. Among these, certain varieties referred to as “Lingzhi Wang” or “Zhu Lingzhi” by indigenous communities are distinguished by their diminutive pilei and slender stipes. Despite their traditional recognition, these species have been subject to morphological confusion. In this study, specimens labeled as “Lingzhi Wang” or “Zhu Lingzhi” were subjected to comprehensive morphological examinations and molecular phylogenetic analyses. The findings reveal that the *Ganoderma* species characterized by small pilei and gracile stipes encompass at least five distinct species. Among these, two are new to science: *G. baisuzhenii* and *G. shennongii*. The remaining three species, *G. bambusicola*, *G. flexipes*, and *G. subflexipes*, have been previously described. Taxonomically, *G. bambusicola* was reported for the first time on the Chinese mainland. This study provides a clearer taxonomic framework for these medicinally significant fungi.

## 1. Introduction

The family Ganodermataceae (Polyporales, Basidiomycota) is a globally distributed taxonomic group comprising fourteen accepted genera: *Amauroderma* Murrill, *Amaurodermellus* Costa-Rezende, Drechsler-Santos & Góes-Neto, *Cristataspora* Robledo & Costa-Rezende, *Foraminispora* Robledo, Costa-Rez. & Drechsler-Santos, *Furtadoella* B.K. Cui & Y.F. Sun, *Ganoderma* P. Karst., Haddowia Steyaert, *Humphreya* Steyaert, Magoderna Steyaert, *Neoganoderma* B.K. Cui & Y.F. Sun, *Sanguinoderma* Y.F. Sun, D.H. Costa & B.K. Cui, *Sinoganoderma* B.K. Cui, J.H. Xing & Y.F. Sun, *Tomophagus* Murrill, and *Trachydermella* B.K. Cui & Y.F. Sun [1,2]. Among these, the genus *Ganoderma*, typified by *G. lucidum* (Curtis) P. Karst., is the most widely recognized. It is characterized by sessile to stipitate basidiomata, double-walled and truncated basidiospores, and its wood-decaying properties [2,3]. According to the Index Fungorum database (http://www.indexfungorum.org/, accessed on 25 January 2025), there are 498 records under *Ganoderma*, although many of these epithets are synonyms [1,4]. In China, more than 40 species of this genus have been accepted to date [1,2,5,6,7,8,9,10].

For more than a century, the highly valued medicinal fungus “Lingzhi” or “Ruizhi”, widely cultivated and utilized in China, including Hainan Island, has been taxonomically classified as *G. lucidum* (Curtis) P. Karst., a species first described in Europe. However, recent studies have revealed that the famous “*G. lucidum*” in China is genetically and taxonomically distinct from the authentic European *G. lucidum*. This Chinese species has been scientifically named *G. lingzhi* S.H. Wu, Y. Cao & Y.C. Dai [10,11]. Despite this, Yao et al. and Du et al. have argued that the correct name for the widely recognized *Ganoderma* species by the public in China should be *G. sichuanense* J.D. Zhao & X.Q. Zhang, with *G. lingzhi* considered a later synonym [12,13]. The scientific nomenclature of this fungus remains a contentious issue, with ongoing debates between *G. lingzhi* and *G. sichuanense*.

*Ganoderma* species, including *G. lingzhi* or *G. sichuanense*, have been used in traditional Chinese medicine for more than 6800 years [14,15,16]. These fungi are celebrated for their rich array of natural bioactive compounds, which exhibit a broad spectrum of pharmacological properties, including immunoregulatory, anti-tumor, anti-fatigue, gut microbiota regulation, and hepatocyte protection activities [17,18,19]. Subtropical and tropical regions of China, particularly Hainan Island, are biodiversity hotspots for *Ganoderma* species [20,21]. In these regions, numerous wild *Ganoderma* species are commercially traded [22], and their fruiting bodies are commonly used by local residents, either soaked in wine or boiled in water, for health preservation and disease treatment [23].

Among the *Ganoderma* species in Hainan Island, certain varieties are locally known as “Lingzhi Wang” (king of *Ganoderma*) or “Zhu Lingzhi” (bamboo host of *Ganoderma*). These species are distinguished by their diminutive pilei and gracile stipes. Due to their high popularity in the region (based on our investigations), they are often subject to morphological confusion, with homonyms (different *Ganoderma* species sharing the same name) and synonyms (the same *Ganoderma* species referred to by different names) being common. To address these taxonomic challenges, this study conducted comprehensive field investigations, key informant interviews, morphological examinations, and molecular phylogenetic analyses to accurately identify and clarify the taxonomy of these species. 

## 2. Materials and Methods

### 2.1. Key Informant Interview Methodology

Key informants were selected based on their expertise and involvement in the collection, trade, or traditional use of *Ganoderma* species on Hainan Island. The interviews were semi-structured, combining open-ended questions with a predefined interview guide to ensure flexibility and depth. The interview guide covered the following topics: background information, traditional knowledge, harvesting and trade practices, cultural significance, and conservation perspectives.

### 2.2. Sample Collection and Morphological Studies

Fresh basidiomata of *Ganoderma* species were collected during the rainfall seasons in southern China, particularly on Hainan Island. Specimens were photographed in situ at their collection sites to document their natural habitat and morphological features. Detailed morphological characteristics, including color, shape, and size, were recorded from fresh samples. The collected specimens were then dried at approximately 60 °C overnight to ensure complete dehydration and were subsequently deposited in the Hainan Biodiversity Science and Technology Museum (FHMU) or the Cryptogamic Herbarium (HKAS) of the Kunming Institute of Botany, Chinese Academy of Sciences. Color descriptions were standardized using the color codes provided by Kornerup and Wanscher [24].

For microscopic analysis, thin sections were prepared freehand using a razor blade and mounted in 5% potassium hydroxide (KOH) solution or Cotton Blue and Melzer’s reagent. These sections were examined under an OLYMPUS CX23 optical microscope to observe and measure microscopic features. All microscopic structures were illustrated freehand based on the observations. The following notations and abbreviations were used in this study: IKI (Melzer’s reagent; IKI– indicates neither amyloid nor dextrinoid reactions); CB (Cotton Blue; CB+ denotes cyanophilous reactions); and basidiospore measurements are expressed as n/m/p, where n = the total number of basidiospores measured from the m basidiomata of p collections. Basidiospore dimensions are presented in the format (a)b–c(d), where b–c represents the range encompassing at least 90% of the measured values, and extreme values (a and d, when present) are given in parentheses. Additional abbreviations include L (mean basidiospore length), W (mean basidiospore width), and Q (quotient of basidiospore length to width, L/W). All measurements were based on a minimum of 20 basidiospores per species.

### 2.3. Molecular Procedures

Genomic DNA was extracted from dried basidiomata samples using the Magnetic Beads Genomic DNA Extraction Kit (Magen, Guangzhou, China) in accordance with the manufacturer’s protocol. The ITS region, including internal transcribed spacers 1 and 2, along with the 5.8S rDNA, was amplified using the primer pair ITS5/ITS4 [25]. Additionally, the partial RNA polymerase second-largest subunit region (*rpb*2) was amplified using primers bRPB2-6F/bRPB2-7.1R [26], and the partial translation elongation factor 1-α (*tef*1) region was amplified using primers EF1-F and EF1-R [27]. All PCR reactions were conducted in 25 μL volumes, containing 13 µL of 2× Taq PCR MasterMix (KANGWEI Company, Guangzhou, China), 2 µL of each primer (10 mM), 2 µL of DNA template, and 8 µL of nuclease-free water. The PCR conditions for all genetic regions included an initial denaturation step at 95 °C for 4 min, followed by 35 cycles of denaturation at 94 °C for 30 s, annealing at specific temperatures (50 °C for ITS, 53 °C for *tef*1, and 52 °C for *rpb*2) for 30 s, extension at 72 °C for 120 s, and a final extension at 72 °C for 7 min. PCR products were visualized on 1% (*w*/*v*) agarose gels, and those exhibiting a bright single band were purified and sequenced using an ABI 3730xl DNA Analyzer (Guangzhou Branch of BGI, Guangzhou, China) with the same primers used for amplification [28]. The accuracy of the newly obtained sequences was confirmed by comparison with sequences available in GenBank [https://www.ncbi.nlm.nih.gov/genbank/ (accessed on 1 February 2025)]. The sequences were assembled and edited using BioEdit v7.0.9.0 [29] and subsequently deposited in GenBank (Table 1).

### 2.4. Dataset Assembly

The phylogenetic analyses were conducted using a sequence dataset comprising three loci: ITS, *rpb*2, and *tef*1. *Foraminispora concentrica* (J. Song, Xiao L. He & B.K. Cui) Y.F. Sun & B.K. Cui was selected as the outgroup, which was suggested by Sun et al. [30]. Additionally, other reference taxa for the phylogenetic analysis were retrieved from GenBank and relevant publications, as detailed in Table 1. To assess potential phylogenetic conflicts among the different gene regions, single-gene phylogenetic trees were constructed for ITS, *rpb*2, and *tef*1 separately using the maximum likelihood (ML) method. The results indicated no significant conflicts among the gene fragments, supporting their combination for further analysis. The sequences of the three loci were aligned using MUSCLE v. 3.6 [31] and subsequently concatenated into a single dataset using Phyutility v. 2.2 [32] for comprehensive phylogenetic analysis.

**Table 1:** Taxa, vouchers, locations, and GenBank accession numbers of DNA sequences used in this study.

			GenBank Accession Nos.			
Taxon	Voucher	Locality	ITS	*rpb*2	*tef*1	Reference
*Cristataspora coffeata*	FLOR 50933	Brazil	KU315204	—	—	[33]
*C. coffeata*	Robledo 3183	Brazil	MN077526	—	MN061695	[34]
*C. coffeata*	Robledo 3182	Brazil	MN077525	—	—	[34]
*C. flavipora*	Robledo 3288	Argentina	MN077521	—	MN061694	[34]
*Foraminispora concentrica*	Cui 16238	Yunnan, SW China	MK119816	MK121504	MK121565	[30]
*F. concentrica*	Cui 16239	Yunnan, SW China	MK119817	MK121506	MK121566	[30]
*Ganoderma acaciicola*	Cui 16815	Australia	MZ354895	MZ245384	—	[2]
*G. acaciicola*	Cui 16814	Australia	MZ354894	MZ245383	—	[2]
*G. acontextum*	JV 0611/21G	Guatemala	KF605667	MG367489	MG367538	[2]
*G. acontextum*	JV 1208/11J	USA	KF605668	MG367490	MG367540	[2]
*G. adspersum*	ITA 39	Italy	EF060011	—	—	[35]
*G. adspersum*	PF263	Italy	JN176908	—	—	Unpublished
*G. alpinum*	Cui 17467	Yunnan, SW China	MZ354912	—	—	[2]
*G. alpinum*	Cui 18402	Xizhang, western China	MZ354910	—	—	[2]
*G. angustisporum*	Cui 13817	Fujian, SE China	MG279170	MG367507	MG367563	[6]
*G. angustisporum*	Cui 14578	Guangdong, southern China	MG279171	—	MG367564	[6]
*G. applanatum*	Cui 14062	Jinlin, NE China	MZ354913	MZ358846	MZ221635	[2]
*G. applanatum*	Cui 14070	Jinlin, NE China	MZ354914	MZ245387	MZ221636	[2]
*G. aridicola*	Dai 12588	South Africa	KU572491	—	KU572502	[36]
*G. aridicola*	GanoTK25	Cameroon	JN105707	—	—	[2]
*G. artocarpicola*	HL173	Yunnan, SW China	ON994239	OP508428	OP508442	[37]
*G. artocarpicola*	HL188	Yunnan, SW China	ON994240	OP508427	OP508441	[37]
*G. australe*	DHCR411	Australia	MF436675	—	MF436677	[38]
*G. australe*	DHCR417	Australia	MF436676	—	MF436678	[38]
*G. austroafricanum*	CBS138724	South Africa	KM507324	—	—	[39]
*G. bambusicola*	Wu 1207-151	Taiwan, SE China	MN957781	LC517944	LC517941	[8]
***G. baisuzhenii***	**N.K. Zeng2080** **(FHMU2334)**	**Hainan, southern China**	**—**	**PP785032**	**PV066218**	**This study**
***G. baisuzhenii***	**N.K. Zeng2519** **(FHMU7350)**	**Hainan, southern China**	**PP663110**	**PP785031**	**PV066219**	**This study**
***G. bambusicola***	**N.K. Zeng1892** **(FHMU1217) R71**	**Hainan, southern China**	**—**	**—**	**PP922172**	**This study**
***G. bambusicola***	**N.K. Zeng1892-1** **(FHM7610)**	**Hainan, southern China**	**—**	**—**	**PP922173**	**This study**
***G. bambusicola***	**N.K. Zeng10386 (FHMU8798)**	**Hainan, southern China**	**PV052368**	**—**	**PV066216**	**This study**
***G. bambusicola***	**N.K. Zeng10387 (FHMU8803)**	**Hainan, southern China**	**PV052369**	**—**	**—**	**This study**
***G. bambusicola***	**N.K. Zeng10388 (FHMU8791)**	**Hainan, southern China**	**PV052370**	**PV066220**	**—**	**This study**
***G. bambusicola***	**N.K. Zeng10340 (FHMU7930)**	**Hainan, southern China**	**PV052371**	**PV066221**	**PV066217**	**This study**
*G. boninense*	WD 2028	Japan	KJ143905	KJ143964	KJ143924	[40]
*G. boninense*	WD 2085	Japan	KJ143906	KJ143965	KJ143925	[40]
*G. brownii*	JV 1105/9J	Australia	MG279159	MG367494	MG367547	[6]
*G. brownii*	JV 0709/109	—	KF605662	MG367495	MG367548	[2,6]
*G. bubalinomarginatum*	Dai 20074	Guangxi, southern China	MZ354926	MZ245388	MZ221637	[2]
*G. bubalinomarginatum*	Dai 20075	Guangxi, southern China	MZ354927	MZ245389	MZ221638	[2]
*G. calidophilum*	MFLU 19-2174	Yunnan, SW China	MN398337	—	—	[3]
*G. calidophilum*	H36	Yunnan, SW China	MW750241	MW839003	MW838997	[1]
*G. carnosum*	JV 8709/8	Czech Republic	KU572493	—	—	[36]
*G. carnosum*	MJ 21/08	Czech Republic	KU572492	—	—	[36]
*G. carocalcareus*	DMC 322	Cameroon	EU089969	—	—	[41]
*G. carocalcareus*	DMC 513	Cameroon	EU089970	—	—	[41]
*G. castaneum*	Dai 16500	Hainan, southern China	MZ354918	MZ245390	MZ221639	[2]
*G. castaneum*	Cui 13893	Hainan, southern China	MZ221640	MZ245391	MZ354919	[2]
*G. casuarinicola*	Dai 16336	Guangdong, southern China	MG279173	MG367508	MG367565	[6]
*G. casuarinicola*	Dai 16337	Guangdong, southern China	MG279174	MG367509	MG367566	[6]
*G. chalceum*	URM80457	Brazil	JX310812	—	—	[42]
*G. chocoense*	QCAM 3123	Ecuador	MH890527	—	—	[43]
*G. chuxiongense*	Cui 17262	Yunnan, SW China	MZ354907	—	—	[2]
*G. cocoicola*	Cui 16791	Australia	MZ354984	MZ245393	MZ221643	[2]
*G. cocoicola*	Cui 16792	Australia	MZ354985	MZ245394	MZ221644	[2]
*G. concinnum*	Robledo 3192 (FCOS)	—	MN077522	—	—	[34]
*G. concinnum*	Robledo 3235 (FCOS)	—	MN077523	—	—	[34]
*G. cupreum*	GanoTK4	Cameroon	JN105701	—	—	Unpublished
*G. cupreum*	GanoTK7	Cameroon	JN105702	—	—	Unpublished
*G. curtisii*	CBS 100131	NC, USA	JQ781848	KJ143966	KJ143926	[11,40]
*G. curtisii*	CBS 100132	NC, USA	JQ781849	KJ143967	KJ143927	[11,40]
*G. destructans*	CMW43670	South Africa	KR183856	—	—	[39]
*G. dianzhongense*	L4331	Yunnan, SW China	MW750237	MZ467043	MW838993	[1]
*G. dianzhongense*	L4969	Yunnan, SW China	MW750240	MZ467044	MW838996	[1]
*G. dorsale*	MVHC 5701	Uruguay	MN191581	—	—	[44]
*G. dorsale*	MVHC 5653	Uruguay	MN191578	—	—	[44]
*G. dunense*	CMW42149	South Africa	MG020248	—	MG020226	[45]
*G. dunense*	CMW42157	South Africa	MG020255	—	—	[45]
*G. ecuadorense*	ASL799	Ecuador	KU128524	—	—	[46]
*G. ecuadorense*	PMC126	Ecuador	KU128525	—	—	[46]
*G. eickeri*	CMW 49692	South Africa	MH571690	—	MH567287	[47]
*G. eickeri*	CMW 50325	South Africa	MH571689	—	MH567290	[47]
*G. ellipsoideum*	GACP14080966	Hainan, southern China	MH106867	—	—	[5]
*G. ellipsoideum*	GACP14080968	Hainan, southern China	MH106868	—	—	[5]
*G. ellipsoideum*	GACP14081228	Hainan, southern China	MH106886	—	—	[5]
*G. enigmaticum*	CMW43669	South Africa	KR183855	—	—	[39]
*G. enigmaticum*	CBS 139792	South Africa	NR132918	—	—	[39]
*G. esculentum*	L4935	Yunnan, SW China	MW750242	MW839004	MW838998	[1]
*G. esculentum*	HL107	Yunnan, SW China	ON994243	OP508424	OP508437	[48]
*G. fallax*	JV 1009/27	USA	KF605655	—	—	[2]
*G. fallax*	JV 0709/39	USA	KF605658	—	—	[2]
*G. flexipes*	Cui 13841	Hainan, southern China	MZ354923	MZ245401	MZ221655	[2]
*G. flexipes*	Cui 13863	Hainan, southern China	MZ354924	MZ245402	MZ221656	[2]
*G. flexipes*	GACP14045450	Hainan, southern China	MH106873	—	—	[5]
*G. flexipes*	Wei5200	China	JN383978	—	—	[49]
***G. flexipes***	**N.K. Zeng2607** **(FHMU3352)**	**Hainan, southern China**	**PP663099**	**—**	**—**	**This study**
***G. flexipes***	**N.K. Zeng2042 (FHMU2329)**	**Hainan, southern China**	**PP663094**	**PP785027**	**PP911339**	**This study**
***G. flexipes***	**N.K. Zeng2616 (FHMU5678)**	**Hainan, southern China**	**PP663101**	**—**	**—**	**This study**
***G. flexipes***	**N.K. Zeng2617 (FHMU5663)**	**Hainan, southern China**	**PP663096**	**—**	**—**	**This study**
***G. flexipes***	**N.K. Zeng2624 (FHMU5681)**	**Hainan, southern China**	**PP663103**	**—**	**—**	**This study**
***G. flexipes***	**N.K. Zeng2627 (FHMU5659)**	**Hainan, southern China**	**PP663098**	**—**	**—**	**This study**
***G. flexipes***	**N.K. Zeng2606 (FHMU3360)**	**Hainan, southern China**	**PP663100**	**—**	**—**	**This study**
***G. flexipes***	**N.K. Zeng2614 (FHMU5672)**	**Hainan, southern China**	**PP663104**	**—**	**—**	**This study**
***G. flexipes***	**N.K. Zeng210 (FHMU2292)**	**Hainan, southern China**	**PP663095**	**—**	**—**	**This study**
***G. flexipes***	**N.K. Zeng2618 (FHMU5661)**	**Hainan, southern China**	**PP663097**	**—**	**—**	**This study**
***G. flexipes***	**N.K. Zeng4561 (FHMU4863)**	**Hainan, southern China**	**PP663106**	**—**	**PP922159**	**This study**
***G. flexipes***	**N.K. Zeng4595 (FHMU4898)**	**Hainan, southern China**	**PP663102**	**—**	**PP922158**	**This study**
***G. flexipes***	**N.K. Zeng2087 (FHMU2337)**	**Hainan, southern China**	**PP663107**	**PP785029**	**PP922162**	**This study**
***G. flexipes***	**N.K. Zeng2085 (FHMU2336)**	**Hainan, southern China**	**PP663108**	**PP785028**	**PP922161**	**This study**
*“G. flexipes”*	Wei5491	Hainan, southern China	JQ781850	KJ143968	—	[11,40]
*“G. flexipes”*	Wei5494	Hainan, southern China	JN383979	—	—	[11]
*G. gibbosum*	Cui 13940	China	MZ354972	MZ245404	MZ221658	[2]
*G. gibbosum*	Cui 14338	China	MZ354969	MZ245405	MZ221659	[2]
*G. guangxiense*	Cui 14453	Guangxi, southern China	MZ354939	MZ245407	MZ221661	[2]
*G. guangxiense*	Cui 14454	Guangxi, southern China	MZ354941	MZ245408	MZ221662	[2]
*G. guixiense*	GXU3457	Guangxi, southern China	OQ788244	PP187389	—	[50]
*G. guixiense*	GXU3709	Guangxi, southern China	OR271986	—	—	[50]
*G. hochiminhense*	MFLU 19-2224	Vietnam	MN398324	—	MN423176	[3]
*G. hochiminhense*	MFLU 19-2225	Vietnam	MN396662	—	MN423177	[3]
*G. hoehnelianum*	GACP14080913	Hainan, southern China	MH106881	—	—	[5]
*G. hoehnelianum*	MFLU 19-2168	Myanmar	MN396316	MN423123	MN423158	[3]
*G. knysnamense*	CMW 47755	South Africa	MH571681	—	MH567261	[47]
*G. knysnamense*	CMW 47756	South Africa	MH571684	—	MH567274	[47]
*G. leucocontextum*	GDGM 40200	Xizang, western China	KF011548	—	—	[51]
*G. lingzhi*	Dai 12479	Anhui, central China	JQ781864	JX029979	JX029975	[11]
*G. lingzhi*	Wu 1006-38	Hubei, eastern China	JQ781858	JX029980	JX029976	[11]
*G. lobatum*	JV 0402/24	—	KF605677	—	—	Unpublished
*G. lobatum*	JV 1212/10J	—	KF605676	—	—	Unpublished
*G. lucidum*	Rivoire 4195	France	KJ143909	KJ143969	—	[40]
*G. lucidum*	K 175217	UK	KJ143911	KJ143971	KJ143929	[40]
*G. magniporum*	Dai 19966	Yunnan, SW China	—	MZ345728	MZ221670	[2]
*G. martinicense*	SWMart08-55	Martinique	KF963256	—	—	[52]
*G. mbrekobenum*	UMN7-3 GHA	Ghana	KX000896	—	—	[46]
*G. mbrekobenum*	UMN7-4 GHA	Ghana	KX000898	—	—	[46]
*G. meredithiae*	UMNFL50	USA	MG654103	—	—	[53]
*G. meredithiae*	UMNFL64	USA	MG654106	MG754863	—	[53]
*G. mexicanum*	MUCL 49453 SW17	Martinique	MK531811	MK531836	MK531825	[54]
*G. mexicanum*	MUCL 55832	Martinique	MK531815	MK531839	MK531829	[54]
*G. mirabile*	Cui 18271	Malaysia	MZ354958	MZ345729	MZ221672	[2]
*G. mirabile*	Cui 18283	Malaysia	MZ354959	MZ345730	MZ221673	[2]
*G. mizoramense*	UMN-MZ4	India	KY643750	—	—	[55]
*G. mizoramense*	UMN-MZ5	India	KY643751	—	—	[55]
*G. multipileum*	CWN 04670 (TNM)	Taiwan, SE China	KJ143913	KJ143972	KJ143931	[40]
*G. multipileum*	Dai 9447 (IFP)	Hainan, southern China	KJ143914	KJ143973	KJ143932	[40]
*G. multipileum*	MFLU 19-2166	Thailand	MN401406	MN423142	MN423172	[3]
*G. mutabile*	Yuan 2289	Yunnan, SW China	JN383977	—	—	[50]
*G. mutabile*	CLZhao 982	Yunnan, SW China	MG231527	—	—	Unpublished
*G. myanmarense*	MFLU 19-2167	Myanmar	MN396330	—	—	[3]
*G. myanmarense*	MFLU 19-2169	Myanmar	MN396330	—	—	[3]
*G. nasalanense*	GACP17060211	Laos	MK345441	—	—	[3]
*G. nasalanense*	GACP17060212	Laos	MK345442	—	—	[49]
*G. neojaponicum*	FFPRI WD-1285	Japan	MN957784	—	—	[8]
*G. neojaponicum*	FFPRI WD-1532	Japan	MN957785	—	—	[8]
*G. nitidum*	JV 1504/73	Hainan, southern China	MZ354933	—	MZ221681	[2]
*G. obscuratum*	Lsh88	Yunnan, SW China	ON994237	—	OP508450	[48]
*G. obscuratum*	Lsh89	Yunnan, SW China	ON994238	—	OP508451	[48]
*G. orbiforme*	Cui 13880	Hainan, southern China	MG279187	MG367523	MG367577	[6]
*G. orbiforme*	Cui 13891	China	MZ354953	MZ345736	MZ221682	[2]
*G. oregonense*	CBS 265.88	USA	JQ781875	KJ143974	KJ143933	[11,40]
*G. oregonense*	CBS 266.88	USA	JQ781876	KJ143975	—	[11,40]
*G. ovisporum*	HKAS123193	Guizhou, SW China	MZ519547	MZ547661	—	[9]
*G. ovisporum*	GACP20071602	Guizhou, SW China	MZ519548	MZ547662	—	[9]
*G. parvulum*	URM83343	Brazil	JQ618246	—	—	[45]
*G. parvulum*	URM80765	Brazil	JX310822	—	—	[45]
*G. pfeifferi*	K(M)120818	UK	AY884185	—	—	Unpublished
*G. philippii*	MFLU 19-2222	Thailand	MN401410	—	MN423174	[3]
*G. philippii*	MFLU 19-2223	Thailand	MN401411	—	MN423175	[3]
*G. phyllanthicola*	L4948	Yunnan, SW China	PP869245	—	—	[37]
*G. phyllanthicola*	HL308	Yunnan, SW China	PP869246	—		[37]
*G. platense*	BAFC384	Argentina	AH008109	—	—	[56]
*G. platense*	BAFC2374	Argentina	AH008110	—	—	[56]
*G. podocarpense*	QCAM 6422	Ecuador	MF796661	—	—	[55]
*G. podocarpense*	JV 1504/126	—	MZ354942	MZ345737	MZ221687	[2]
*G. polychromum*	UMNOR3	USA	MG654204	—	MG754744	[53]
*G. polychromum*	MS343OR	USA	MG654197	—	MG754743	[53]
*G. puerense*	Dai 20427	Yunnan, SW China		MZ345738	MZ221688	[2]
*G. ramosissium*	xsd08085	—	FJ478127	—	—	[2]
*G. ramosissium*	xsd08032	—	EU918700	—	—	[2]
*G. ravenelii*	MS187FL	USA	MG654211	MG754865	MG754745	[53]
*G. ravenelii*	151FL	USA	MG654208	—	—	[53]
*G. resinaceum*	BCRC 36147	Netherlands	KJ143916	—	KJ143934	[40]
*G. resinaceum*	BR 4150	France	KJ143915	—	—	[40]
*G. ryvardenii*	HKAS58053	Cameroon	HM138671	—	—	[57]
*G. ryvardenii*	HKAS 58054	Cameroon	HM138672	—	—	[57]
*G. sanduense*	SA18012501	Guizhou, SW China	MK345450	—	—	[58]
*G. sanduense*	L4906	Yunnan, SW China	ON994251	OP508430	OP508444	[48]
*G. sessile*	JV 1209/9	USA	KF605629	—	KJ143936	[40]
*G. sessile*	V 1209/27	USA	KF605630	KJ143976	KJ143937	[40]
*G. shanxiense*	BJTC FM423	Shanxi, northern China	MK764268	MK783940	MK783937	[59]
*G. shanxiense*	HSA 539	Shanxi, northern China	MK764269	MK789681	—	[59]
***G. shennongii***	**N.K. Zeng203 (FHMU2290)**	**Hainan, southern China**	**PP663109**	**—**	**—**	**This study**
*G. sichuanense*	HMAS 42798	Sichuan, SW China	JQ781877	—	—	[11]
*G. sichuanense*	Cui 7691	Guangdong, southern China	JQ781878	—	—	[11]
*G. sinense*	Cui 14526	Guangxi, southern China	MZ354961	MZ345743	MZ221694	[2]
*G. sinense*	Cui 14461	China	MZ354963	MZ345744	MZ221695	[2]
*G. steyaertanum*	II-121-1	Indonesia	KJ654427	—	—	[60]
*G. steyaertanum*	6-WN-15(M)-A	Indonesia	KJ654459	—	—	[60]
*G. suae*	L4651	Yunnan, SW China	PP869243	PP894784	PP894782	[37]
*G. suae*	L4817	Yunnan, SW China	PP869244	—	PP894783	[37]
*G. subangustisporum*	Cui 18592	Yunnan, SW China	MZ354981	—	MZ221697	[2]
*G. subangustisporum*	Cui 18593	Yunnan, SW China	MZ354982	—	MZ221698	[2]
*G. subellipsoideum*	Cui 18241	Malaysia	—	—	MZ221701	[2]
*G. subellipsoideum*	Cui 18325	Malaysia	—	—	MZ221702	[2]
***G. subflexipes***	**HKAS81926-3**	**Fujian, SE China**	PP465553	**—**	**PP922169**	[61], this study
***G. subflexipes***	**N.K. Zeng1893-2 (FHMU7611)**	**Hainan, southern China**	**—**	**—**	**PP922171**	This study
***G. subflexipes***	**HKAS80249**	**Fujian, SE China**	**—**	**—**	**PP922168**	This study
***G. subflexipes***	**N.K. Zeng4086** **(FHMU3731)**	**Guangdong, southern China**	**—**	**—**	**PP922165**	This study
***G. subflexipes***	**N.K. Zeng1455** **(FHMU2320)**	**Fujian, SE China**	PP465552	**PP785030**	**PP922167**	[61], this study
***G. subflexipes***	**N.K. Zeng4114** **(FHMU5725)**	**Guangdong, southern China**	PP465551	**—**	**PP922164**	[61], this study
***G. subflexipes***	**HKAS79603**	**Guangdong, southern China**	PP465550	**—**	**PP922166**	[61], this study
***G. subflexipes***	**HKAS81926-1**	**Hainan, southern China**	PP465549	**—**	**PP922163**	[61], this study
*G. subflexipes*	Cui 17257	Guangdong, southern China	MZ354922	MZ245396	MZ221646	[2]
*G. sublobatum*	Cui 16804	Australia	MZ354973	MZ345747	MZ221704	[2]
*G. sublobatum*	Cui 16806	Australia	MZ354974	—	MZ221705	[2]
*G. thailandicum*	HKAS 104640	Thailand	MK848681	MK875831	MK875829	[62]
*G. thailandicum*	HKAS 104641	Thailand	MK848682	MK875832	MK875830	[62]
*G. tongshanense*	Cui 17168	Hubei, central China	MZ354975	—	MZ221706	[2]
*G. tropicum*	BCRC 37122	Taiwan, SE China	EU021457	—	—	[63]
*G. tropicum*	GACP1408 1518	Hainan, southern China	MH106884	—	—	[5]
*G. tsugae*	UMNMI20	USA	MG654324		MG754764	[64]
*G. tsugae*	UMNMI30	USA	MG654326	MG754871	MH025362	[64]
*G. tuberculosum*	GVL-21	Mexico	MT232639	—	—	[65]
*G. tuberculosum*	GVL-40	Mexico	MT232634	—	—	[65]
*G. weberianum*	GanoTK17	Cameroon	JN105705	—	—	[66]
*G. weixiensis*	YL02	Yunnan, SW China	MK302445	—	MK302443	[7]
*G. weixiensis*	YL01	Yunnan, SW China	MK302444	—	MK302442	[7]
*G. wiiroense*	UMN-21-GHA	Ghana	KT952363	—	—	[67]
*G. wiiroense*	UMN-20-GHA	Ghana	KT952361	—	—	[67]
*G. williamsianum*	Dai 17790	Singapore	MZ354947	—	—	[2]
*G. williamsianum*	Dai 16809	Thailand	MG279183	MG367535	MG367588	[6]
*G. yunlingense*	Cui 16288	Yunnan, SW China	MZ354915	—	MZ221718	[2]
*G. yunlingense*	Cui 17043	Yunnan, SW China	MZ354916	—	MZ221719	[2]
*G. yunnanense*	HL45	Yunnan, SW China	ON994235	OP508422	OP508436	[48]
*G. yunnanense*	L4812	Yunnan, SW China	ON994236	OP508429	OP508443	[48]
*G. zonatum*	FL-02	USA	KJ143921	KJ143979	KJ143941	[40]
*G. zonatum*	FL-03	USA	KJ143922	KJ143980	KJ143942	[40]

New sequences are shown in bold. SW: southwestern; NE: northeastern; SE: southeastern.

### 2.5. Phylogenetic Analyses

The phylogenetic tree based on the combined dataset (ITS + *rpb*2 + *tef*1) was reconstructed using both maximum likelihood (ML) and Bayesian inference (BI) methods. For the ML analysis, tree generation and bootstrap analyses were conducted using RAxML 7.2.6 [68], with 1000 bootstrap replicates integrated into an ML search. Bayesian analysis was performed using MrBayes 3.1 [69], employing the Markov Chain Monte Carlo (MCMC) technique. The substitution models for the combined dataset were determined using MrModeltest 2.3 [70], with the best-fit models identified as HKY + I + G for ITS and GTR + I + G for both *rpb*2 and *tef*1. The Bayesian analysis of the combined nuclear dataset (ITS + *rpb*2 + *tef*1) was run for 60 million generations, with trees sampled every 1000 generations. The first 25% of the sampled trees were discarded as burn-in, and a majority consensus tree was constructed from the remaining trees. Bayesian posterior probabilities (PPs) were calculated for the consensus tree. Branches with ML bootstrap values ≥ 70% and Bayesian posterior probabilities (PPs) ≥ 0.95 were considered to have significant support.

## 3. Results

### 3.1. Molecular Data

**Figure 1:**
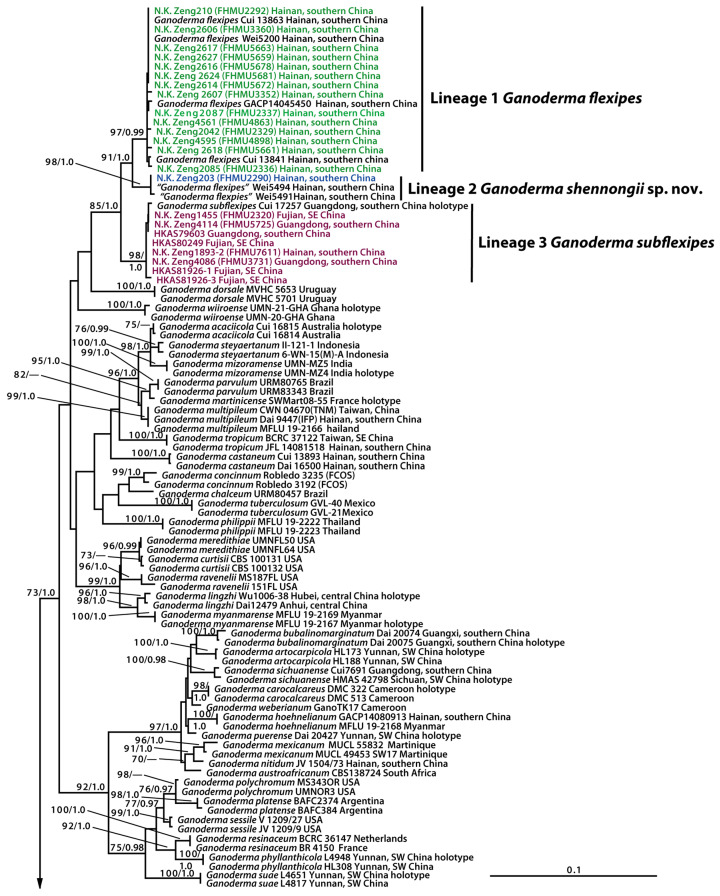

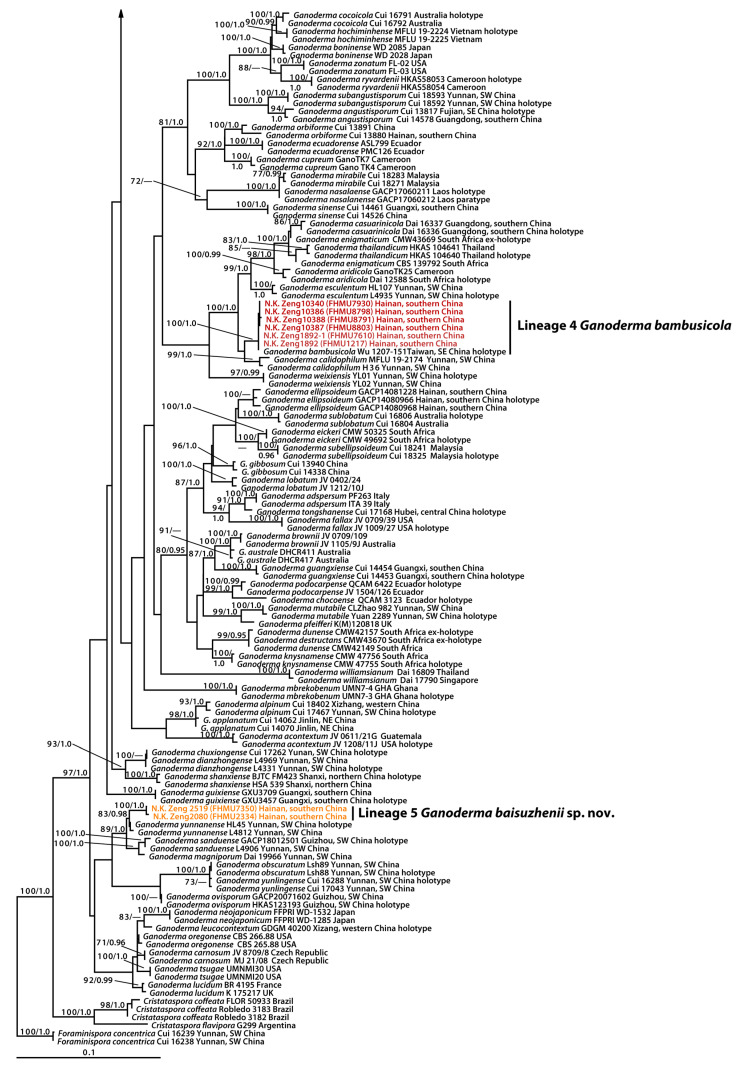
Phylogram for *Ganoderma* species generated from maximum likelihood analysis of ITS, *rpb*2*,* and *tef*1 sequence dataset using RAxML. BS ≥ 70% and PP ≥ 0.95 are indicated above or below the branches as RAxML BS/PP.

The combined dataset (ITS + *rpb*2 + *tef*1) comprised 224 sequences with 2032 nucleotide sites. A phylogram generated using RAxML, displaying branch lengths and support values, is presented in Figure 1. The tree topologies inferred from maximum likelihood (ML) and Bayesian inference (BY) analyses were identical, although slight differences in statistical support were observed (Figure 1). Based on the combined dataset, our newly collected *Ganoderma* specimens with diminutive pilei and gracile stipes were grouped into five distinct lineages (Figure 1). Lineage 1, with strong statistical support (BS = 97%, PP = 0.99), included four collections of *G. flexipes* Pat. and 14 newly collected specimens (FHMU2292, FHMU2329, FHMU2336, FHMU2337, FHMU3352, FHMU3360, FHMU4863, FHMU4898, FHMU5659, FHMU5661, FHMU5663, FHMU5672, FHMU5678, and FHMU5681). Lineage 2, also with strong statistical support (BS = 98%, PP = 1.0), consisted of two collections labeled as *G. flexipes* (Wei5491 and Wei5494) and one newly collected specimen (FHMU2290). Lineage 3, with strong statistical support (BS = 98%, PP = 1.0), included the holotype of *G. subflexipes* B.K. Cui, J.H. Xing & Y.F. Sun and eight newly collected specimens (FHMU2320, FHMU3731, FHMU5725, FHMU7611, HKAS79603, HKAS80249, HKAS81926-1, and HKAS81926-3). Lineage 4, with robust statistical support (BS = 100%, PP = 1.0), grouped the holotype of *G. bambusicola* Sheng H. Wu, C.L. Chern & T. Hatt. with six new specimens (FHMU1217, FHMU7610, FHMU7930, FHMU8791, FHMU8798, and FHMU8803). Finally, Lineage 5, also with strong statistical support (BS = 100%, PP = 1.0), comprised two newly collected specimens (FHMU2334 and FHMU7350).

### 3.2. Taxonomy

Based on morphological examinations and molecular phylogenetic analyses, our new *Ganoderma* collections from the southern region of China, particularly Hainan Island, were identified as five distinct taxa. Among these, three were recognized as *G. bambusicola*, *G. flexipes*, and *G. subflexipes*, while the remaining two represented novel species. In accordance with local tradition, *G. baisuzhenii*, *G. flexipes*, and *G. shennongii* are commonly known as “Lingzhi Wang”, whereas *G. bambusicola* and *G. subflexipes* are referred to as “Zhu Lingzhi”. Detailed morphological descriptions of these five species are provided in the following sections.

**Figure 2:**
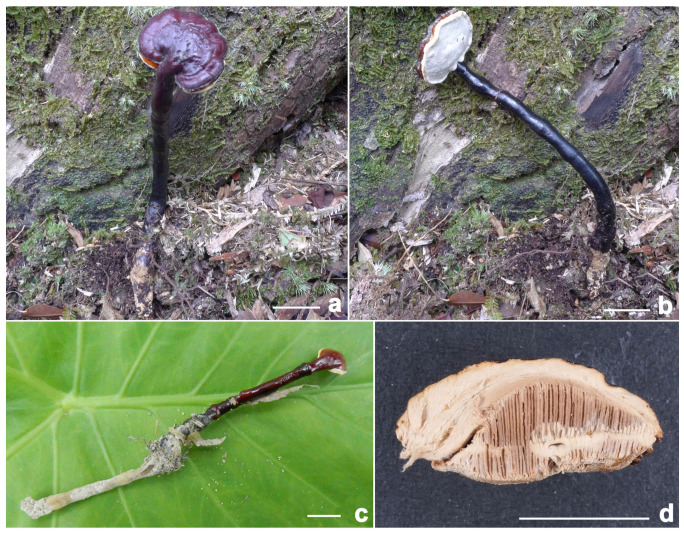
*Ganoderma baisuzhenii* (**a**–**c**) Basidiomata [(**a**,**b**) from FHMU2334, holotype; (**c**) from FHMU7350]. (**d**) Section of pileus (FHMU2334). Scale bars = 1 cm. Photos by N.K. Zeng.

**Figure 3:**
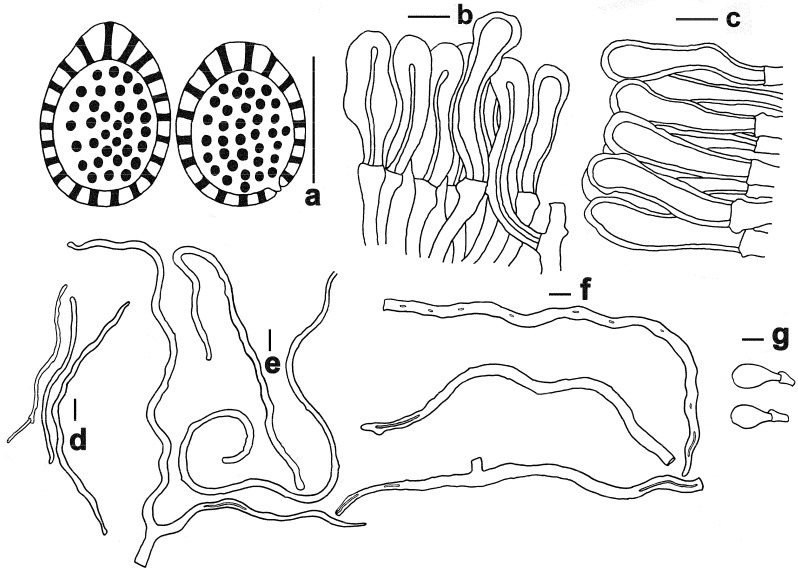
Microscopic features of *Ganoderma baisuzhenii* (FHMU 2334, holotype). (**a**) Basidiospores. (**b**) Pileipellis. (**c**) Stipitipellis. (**d**) Generative hyphae. (**e**) Skeletal hyphae. (**f**) Binding hyphae. (**g**) Basidioles. Scale bars = 10 μm. Drawings by R. Tian.

***Ganoderma baisuzhenii*** N.K. Zeng, R. Tian & Zhi Q. Liang, sp. nov. (Figure 2 and Figure 3)

**MycoBank:** MB854442

**Etymology:** “*baisuzhenii”* is given in honor of Su-Zhen Bai, an ancient Chinese mythical figure who risked her life in search of Lingzhi to save her husband. 

**Diagnosis:** It differs from the closest species of *Ganoderma* by a brownish-red to dark brownish-red pileus, a nearly white context, large pores, a pore surface that was initially yellowish, fading to white with age, and relatively large basidiospores, and it grows on decaying hardwood (underground). 

**Holotype:** CHINA. Hainan Province: Changjiang County, Bawangling of Hainan Tropical Rainforest National Park, elev. 850 m, 28 June 2015, N.K. Zeng2080 (FHMU2334).

**Description:** Basidiomata annual, dorso-laterally stipitate, corky. Pilei up to 2.6 cm diameter and 0.9 cm thick, solitary, sub-reniform to reniform, or flabelliform. Pileal surface brownish-red (8E8) to dark brownish-red (8F8), strongly laccate, glabrous, with concentric furrows and inconspicuously radial rugose; margin obtuse, entire, incurved. Pore surface yellowish when young, then white, turning yellowish-brown (4C5) when injured; pore 2–3 per mm, subcircular, circular or angular; dissepiments slightly thick to moderately thick, entire. Context up to 0.5 cm thick, nearly white (4A3), hard woody. Tube up to 0.7 cm long, brown (5C5), indistinctively stratified. Stipe up to 10 cm long and 0.65 cm diameter, subcylindrical, solid, woody; surface brownish-black (8F7), glabrous to bumpy, laccate.

Hyphal system trimitic; generative hyphae with clamp connections; all hyphae IKI –, CB +; tissues darkening in KOH. Generative hyphae in context 1–3.5 µm diameter, colorless, thin-walled; skeletal hyphae in context 3–6.5 µm diameter, yellowish-brown, thick-walled with a wide to narrow lumen or sub-solid, seldom branched; binding hyphae in context 2.5–4 µm diameter, pale yellow, thin to slight thick-walled, sub-solid to solid, branched and flexuous. Generative hyphae in tubes 2.5–4 µm diameter, colorless, thin-walled; skeletal hyphae in tubes 3–6.5 µm diameter, yellowish-brown, usually thick-walled, solid to sub-solid, rarely branched; binding hyphae in tube 2–4.7 µm diameter, pale yellow, thin to slight thick-walled, sub-solid to solid, branched and flexuous. Pileipellis composed of clamped generative hyphae, thick-walled to sub-solid; apical cells 20–45 × 4–12 μm, clavate, slightly inflated, yellowish-brown, forming a regular palisade. Stipitipellis composed of clamped generative hyphae, thick-walled to sub-solid; apical cells 25–40 × 6–10 μm, clavate, slightly inflated, grayish-yellow, forming a regular palisade. Cystidia and cystidioles absent. Basidioles 15–23 × 10–13.5 μm, clavate, colorless, thin-walled. Basidia not observed. Basidiospores (11–) 11.5–14 (–15) × (7–) 8–10 μm, L = 13.13 μm, W = 9.35 μm, Q = 1.40 (n = 20/1/1, with myxosporium), 9–11 (–11.5) × 6–8 (–8.5) μm, L = 9.9 μm, W = 7.25 μm, Q = 1.37 (n = 20/1/1, without myxosporium), ellipsoid to broadly ellipsoid, pale yellowish-brown to brown, IKI –, CB +, double-walled with distinctly thick walls, exospore wall smooth, endospore wall with dense spinules.

**Habitat:** Solitary or gregarious, growing on decaying hardwood (often underground) of fagaceous trees, particularly those of the genus *Cyclobalanopsis*. 

**Known distribution:** Southern China (Hainan Province). 

**Additional specimen examined:** CHINA. Hainan Province: Yinggeling of Hainan Tropical Rainforest National Park, elev. 750 m, 3 August 2015, N.K. Zeng2519 (FHMU7350). 

**Notes:** *Ganoderma baisuzhenii*, commonly referred to as “Lingzhi Wang” on Hainan Island in tropical China, has been frequently misidentified as *G. flexipes* (based on our investigations). *Ganoderma flexipes* can be distinguished from *G. baisuzhenii* by its richer red pileus, white pore surface, brown pileal context, and smaller basidiospores (see details below). Morphologically and phylogenetically, *G. baisuzhenii* is closely related to *G. magniporum* J.D. Zhao & X.Q. Zhang, *G. sanduense* Hapuar., T.C. Wen & K.D. Hyde, and *G. yunnanense* Jun He & Shu H. Li. However, these species exhibit distinct characteristics: *G. magniporum*, originally described in Guangxi in southern China, features a blackish-brown to black pileus, a brown context, and smaller basidiospores measuring 8.7–10.4 × 5.2–7 μm [71]; *G. sanduense*, first described in Guizhou in southwestern China, is characterized by a reddish-black to brownish-black pileus, smaller pores, and a brown to dark brown context [58]; and *G. yunnanense*, originally described in Yunnan in southwestern China, has smaller pores (4–6 per mm), a white pore surface, and smaller basidiospores measuring 9–12 × 7–8 μm [48].

**Figure 4:**
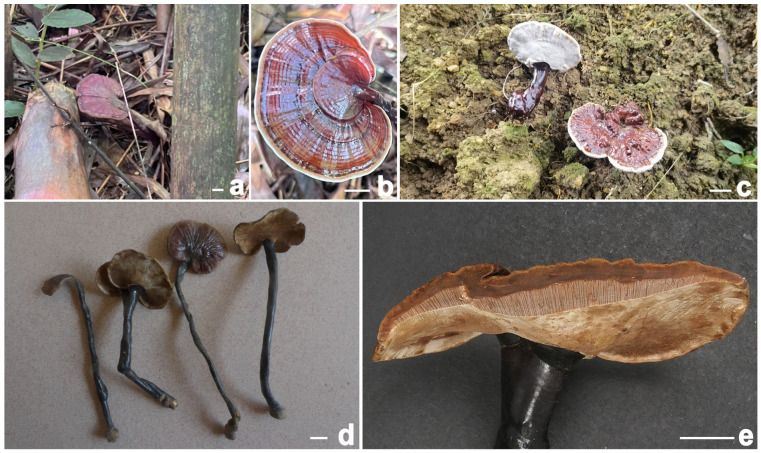
*Ganoderma bambusicola* (**a**–**d**) Basidiomata [(**a**,**b**) from FHMU8798; (**c**) from FHMU7930, cultivated fruit bodies; (**d**) from FHMU1217]. (**e**) Section of pileus (FHMU1217). Scale bars = 1 cm. Photos: (**a**,**b**) by Y. Liu and (**c**–**e**) by N.K. Zeng.

**Figure 5:**
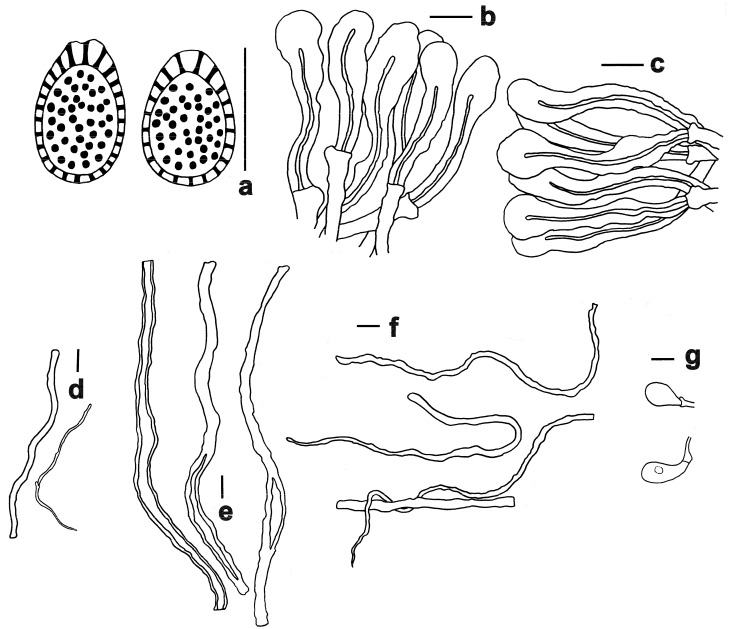
Microscopic features of *Ganoderma bambusicola* (FHMU1217). (**a**) Basidiospores. (**b**) Pileipellis. (**c**) Stipitipellis. (**d**) Generative hyphae. (**e**) Skeletal hyphae. (**f**) Binding hyphae. (**g**) Basidioles. Scale bars = 10 μm. Drawings by R. Tian.

***Ganoderma bambusicola*** Sheng H. Wu, C.L. Chern & T. Hatt. Phytotaxa 456(1): 79, 2020 (Figure 4 and Figure 5)

**MycoBank:** MB835651

**Description:** Basidiomata annual, dorso-laterally stipitate, woody. Pilei up to 7 cm diameter and 2 mm thick, solitary, applanate, sub-reniform to reniform, or flabelliform. Pileal surface brownish-black (8F7–8F8), reddish-black (10F8) to purplish-brown (12E6–12E7), laccate, glabrous, with conspicuously or obscurely concentric furrows and strongly radial rugose; margin obtuse, entire, slightly incurved. Pore surface white (2B2–2B3), turning yellowish-brown (5D5–5D6) when injured; pores 4–6 per mm, subcircular to circular or angular; dissepiments thin, mostly entire. Context up to 0.3 cm thick, dark brown (5E5–5E8), fibrous to corky. Tubes up to 0.4 cm long, grayish-yellow (5C3–5C4), indistinctively stratified. Stipe up to 15 cm long and 1.5 cm diameter, cylindrical to subcylindrical, solid, woody; surface blackish (7F7), glabrous to bumpy, laccate.

Hyphal system trimitic; generative hyphae with clamp connections; all hyphae IKI –, CB +; tissues darkening in KOH. Generative hyphae in context 2–4 µm diameter, nearly colorless, thin-walled; skeletal hyphae in context 4–7 µm diameter, grayish-yellow, thick-walled with a medium to narrow lumen or sub-solid, rarely branched; binding hyphae in context 3–5 µm diameter, light orange, slightly thick-walled, sub-solid, frequently branched. Generative hyphae in tubes 3–6 μm diameter, colorless, thin-walled; skeletal hyphae in tubes grayish-yellow, thick-walled with a narrow lumen to sub-solid, frequently arboriform and flexuous; binding hyphae in tubes 1.5–3.5 μm diameter, light orange, thick-walled, branched and flexuous. Pileipellis composed of clamped generative hyphae, thick-walled to sub-solid; apical cells 30–64 × 6–14 μm, clavate, slightly inflated, yellowish-brown, forming a regular palisade. Stipitipellis composed of clamped generative hyphae, thick-walled to sub-solid; apical cells 35–50 × 7–15 μm, clavate, slightly inflated, yellowish-brown, forming a regular palisade. Cystidia and cystidioles absent. Basidioles 17–24.5 × 9–13 μm, clavate, colorless, thin-walled. Basidia not observed. Basidiospores 9.5–12.5 × 5–7 μm, L = 11.13 μm, W = 6.32 μm, Q = 1.76 (n = 160/8/7, with myxosporium), 7–11 × 4–5.5 μm, L = 9.03 μm, W = 5.08 μm, Q = 1.78 (n = 160/8/7, without myxosporium), ellipsoid to broadly ellipsoid, pale yellowish-brown to brown, IKI–, CB+, double-walled with distinctly thick walls, exospore wall smooth, endospore wall with dense spinules.

**Habitat:** Solitary or gregarious, occurring on dead roots of bamboo, particularly those of *Dendrocalamus latiflorus* Munro.

**Known distribution:** Southern (Hainan Province) and southeastern (Taiwan Province) China, Laos, and Myanmar [8]. 

**Specimens examined:** CHINA. Hainan Province, Haikou City, bought from market, 14 January 2015, N.K. Zeng1892 (FHMU1217); same location and date, N.K. Zeng1892-1 (FHMU7610); Baisha County, Qingsong Town, Edible and Medicinal Fungi Cultivation Base, 2 October 2024, N.K. Zeng10340 (FHMU7930); Baisha County, Nankai Town, elev. 520 m, 7 October 2024, N.K. Zeng10386 (FHMU8798); same location and date, N.K. Zeng10387 (FHMU8803); same location and date, N.K. Zeng10388 (FHMU8791). 

**Notes:** *Ganoderma bambusicola* was originally described in Taiwan in southeastern China [8]. Taxonomically, this species represents a newly recorded addition to the fungal flora of the Chinese mainland. Commonly referred to as “Zhu Lingzhi” on Hainan Island in tropical China, it has been successfully cultivated (based on our investigations). This species is characterized by a brownish-black, reddish-black to purplish-brown pileus, relatively small pores, a white pore surface, and a dark brown context. It is typically found growing on dead bamboo roots.

**Figure 6:**
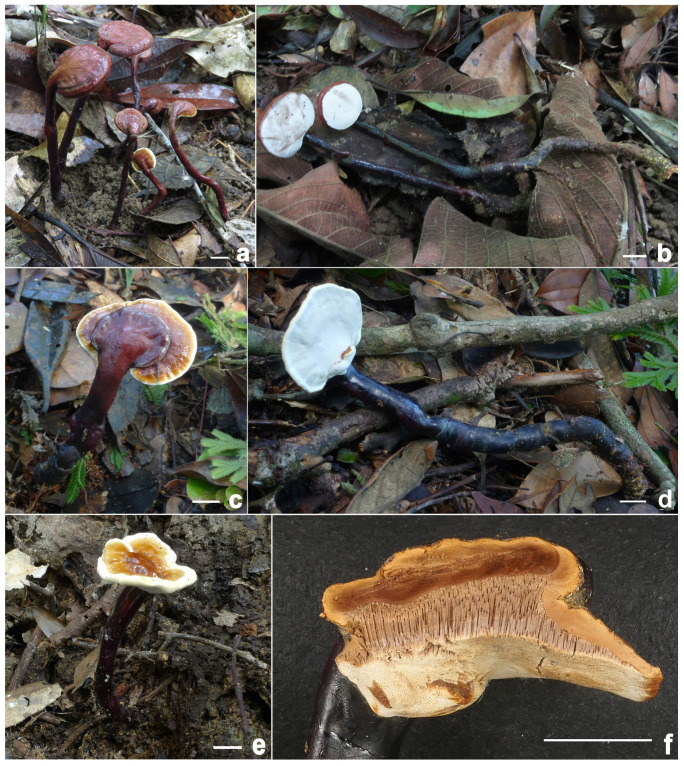
*Ganoderma flexipes* (**a**–**e**) Basidiomata [(**a**,**b**) from FHMU2985; (**c**,**d**) from FHMU2329; (**e**) from FHMU2337]. (**f**) Section of pileus (FHMU2329). Scale bars = 1 cm. Photos by N.K. Zeng.

**Figure 7:**
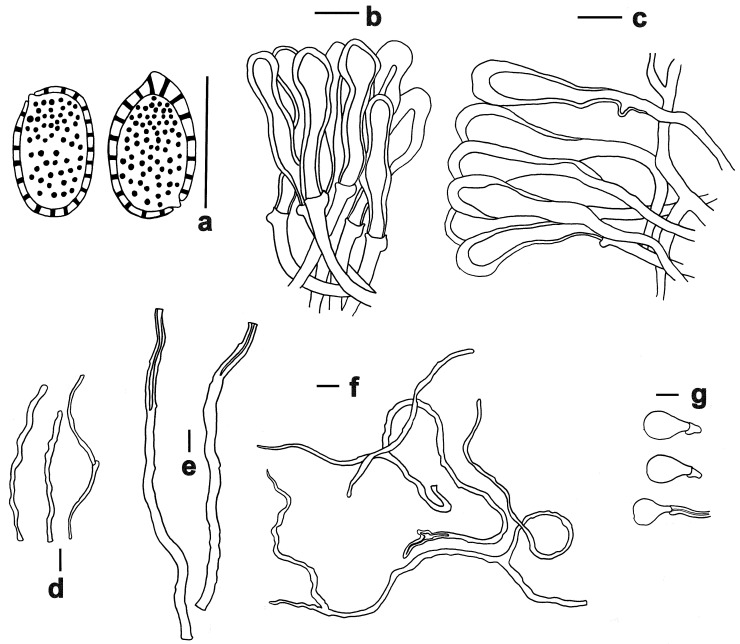
Microscopic features of *Ganoderma flexipes* (FHMU2985). (**a**) Basidiospores. (**b**) Pileipellis. (**c**) Stipitipellis. (**d**) Generative hyphae. (**e**) Skeletal hyphae. (**f**) Binding hyphae. (**g**) Basidioles. Scale bars = 10 μm. Drawings by R. Tian.

***Ganoderma flexipes*** Pat., Bull. Soc. mycol. Fr. 23(2): 75, 1907 (Figure 6 and Figure 7)

≡ *Fomes flexipes* (Pat.) Sacc. & Traverso, Syll. fung. (Abellini) 19: 710, 1910

≡ *Polyporus flexipes* (Pat.) Lloyd, Mycol. Writ. 3 (Syn. Stip. Polyporoids): 104, 1912

= *Ganoderma atrum* J.D. Zhao et al., Acta Microbiol. Sin. 19: 268, 1979

= *Ganoderma hainanense* J.D. Zhao et al., Acta Microbiol. Sin. 19: 269, 1979

= *Ganoderma parviungulatum* J.D. Zhao & X.Q. Zhang, Acta Mycol. Sin. 5: 88, 1986

**MycoBank:** MB249905

**Description:** Basidiomata annual, dorso-laterally, sometimes centrally stipitate, corky. Pilei solitary, up to 5 cm diameter and 0.6 cm thick, sub-reniform to reniform, subflabellate to flabellate, shell-like or circular. Pileal surface brownish-red (8E5–8E6) to reddish, strongly laccate, glabrous when young, then bumpy, with obvious concentric furrows and slightly radial rugose; margin obtuse, entire, slightly incurved. Pore surface white, turning brownish (5D5–5D6) when injured; pore 3–4 per mm, subcircular, circular or angular; dissepiments slightly thick, entire. Context up to 3 mm thick, upper layer yellowish-brown (5D5–5D6), lower layer brown to dark brown (8E8), fibrous to corky. Tubes pale brown (5B3–5B4), indistinctively stratified, up to 0.6 cm long. Stipe up to 17.5 cm long and 0.6 cm diameter, flattened to subcylindrical, solid, woody; surface brownish-red (8E8) to reddish-black (8F8), glabrous to bumpy, laccate. 

Hyphal system trimitic; generative hyphae with clamp connections; all hyphae IKI–, CB+; tissues darkening in KOH. Generative hyphae in context 1–2.5 µm diameter, colorless, thin-walled; skeletal hyphae in context 4–6 µm diameter, yellowish-brown, thick-walled with a wide to narrow lumen or sub-solid, arboriform and flexuous; binding hyphae in context 4–6 µm diameter, colorless, thick-walled, branched and flexuous. Generative hyphae in tubes 1–3 μm diameter, colorless, thin-walled; skeletal hyphae in tubes 2.5–5 μm diameter, colorless, sub-solid, arboriform and flexuous; binding hyphae in tubes 1–3 μm diameter, colorless, thick-walled, branched and flexuous. Pileipellis composed of clamped generative hyphae, thick-walled; apical cells 30–50 × 5–13 µm, clavate, slightly inflated, yellowish-brown or brownish-orange, forming a regular palisade. Stipitipellis composed of clamped generative hyphae, thick-walled to sub-solid; apical cells 32–50 × 5–12 μm, clavate, slightly inflated, pale brown or brownish-orange, forming a regular palisade. Cystidia and cystidioles absent. Basidioles 17.5–23 × 7–11.5 μm, clavate, colorless, thin-walled. Basidia not observed. Basidiospores 8.5–12 × 5.5–7.5 μm, L = 10.15 μm, W = 6.45 μm, Q = 1.57 (n = 80/4/3, with myxosporium); 6.5–9 × 4–6 μm, L = 7.59 μm, W = 5.2 μm, Q = 1.46 (n = 80/4/3, without myxosporium), ellipsoid, not obviously truncated, light yellow (4A5–4A6) to pale yellowish-brown (5C5–5C6), IKI –, CB +, double-walled with moderately thick walls, exospore wall smooth, endospore wall with dense spinules.

**Habitat:** Solitary or gregarious, occurring on decaying hardwood (often underground) of fagaceous trees, particularly those of *Quercus patelliformis* Chun.

**Known distribution:** Southern (Hainan and Guangdong Provinces) [2], southeastern (Taiwan Province) [72], and southwestern (Yunnan Province) China [3], Vietnam, India, Laos, Nepal, Pakistan, and Myanmar [3,58].

**Specimens examined:** CHINA. Hainan Province: Wuzhishan of Hainan Tropical Rainforest National Park, elev. 1200 m, 30 May 2009, N.K. Zeng210 (FHMU2292); Jianfengling of Hainan Tropical Rainforest National Park, elev. 900 m, 30 June 2015, N.K. Zeng2087 (FHMU2337); same location, 27 June 2018, N.K. Zeng3420 (FHMU2985); same location, 10 August 2020, N.K. Zeng4561 (FHMU4863); same location, 11 August 2020, N.K. Zeng4595 (FHMU4898); Bawangling of Hainan Tropical Rainforest National Park, elev. 950 m, 28 June 2015, N.K. Zeng2042 (FHMU2329); same location and date, N.K. Zeng2085 (FHMU2336); Yinggeling of Hainan Tropical Rainforest National Park, elev. 800 m, 5 August 2015, N.K. Zeng2606 (FHMU3360); same location and date, N.K. Zeng2607 (FHMU3352); Ledong County, Jianfeng Town, 16 November 2015, bought from the market, N.K. Zeng2614 (FHMU5672); same location and date, N.K. Zeng2616 (FHMU5678); same location and date, N.K. Zeng2617 (FHMU5663); same location and date, N.K. Zeng2618 (FHMU5661); same location and date, N.K. Zeng2624 (FHMU5681); same location and date, N.K. Zeng2627 (FHMU5659).

**Notes:** *Ganoderma flexipes* was originally described in Vietnam [73] and has since been reported in several other regions, including China, India, Laos, Nepal, Pakistan, and Myanmar [3,58,72,74,75]. In Hainan Island, tropical China, this species is commonly referred to as “Lingzhi Wang”. It is characterized by a brownish-red to reddish pileus, a white pore surface, and a yellowish-brown to dark brown context. It typically grows on the decaying hardwood (often underground) of fagaceous trees.

**Figure 8:**
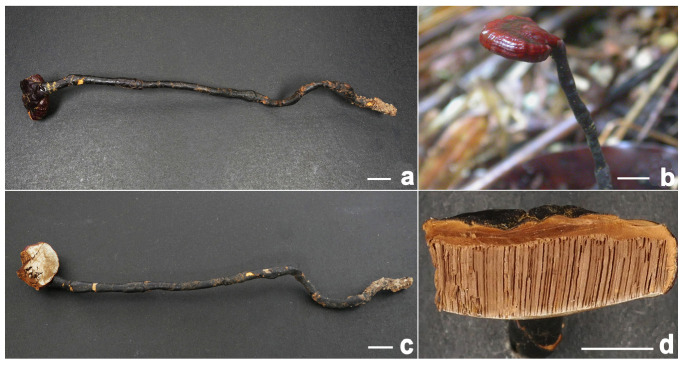
*Ganoderma shennongii* (FHMU2290, holotype) (**a**–**c**) Basidiomata. (**d**) Section of pileus. Scale bars = 1 cm. Photos by N.K. Zeng.

**Figure 9:**
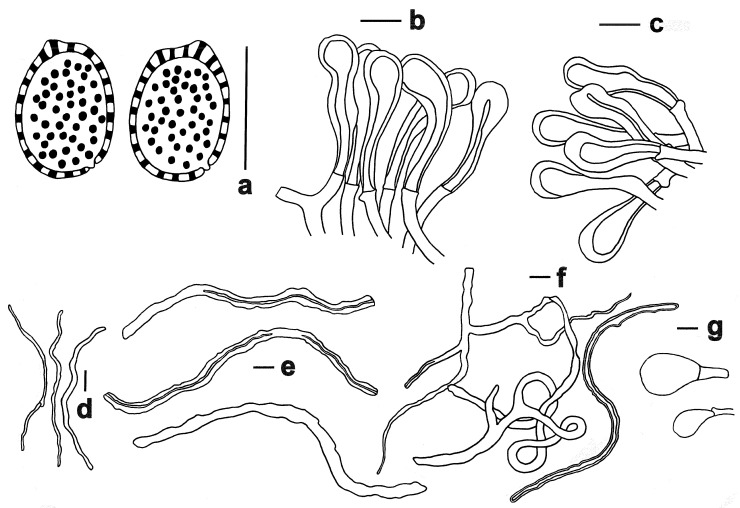
Microscopic features of *Ganoderma shennongii* (FHMU2290, holotype). (**a**) Basidiospores. (**b**) Pileipellis. (**c**) Stipitipellis. (**d**) Generative hyphae. (**e**) Skeletal hyphae. (**f**) Binding hyphae. (**g**) Basidioles. Scale bars = 10 μm. Drawings by R. Tian.

***Ganoderma shennongii*** N.K. Zeng, R. Tian & Zhi Q. Liang, **sp. nov.** (Figure 8 and Figure 9)

**MycoBank:** MB854441

**Etymology:** “s*hennongii”* is given in honor of our ancestor represented by Shennong who dared to taste hundreds of herbs including *Ganoderma* spp.

**Diagnosis:** It differs from the closest species of *Ganoderma* by a relatively small basidioma, a dark reddish pileus, a white pore surface, relatively small pores, and a yellowish-brown to brown context, and it grows on decaying dead wood (underground).

**Holotype:** CHINA. Hainan Province, Diaoluoshan of Hainan Tropical Rainforest National Park, elev. 950 m, 28 May 2009, N.K. Zeng203 (FHMU2290). 

**Description:** Basidiomata annual, dorso-laterally stipitate, corky. Pilei up to 1.8 cm diameter and 0.6 cm thick, solitary, sub-reniform to reniform, or subflabellate to flabellate. Pileal surface dark reddish (9E5–9E6), strongly laccate, glabrous, with conspicuously concentric furrows and slightly radial rugose; margin obtuse, entire, incurved. Pore surface white, turning brown (5C5–5C6) when injured; pores 4–5 per mm, subcircular to circular or angular; dissepiments slightly thick to moderately thick, entire. Context up to 0.1 cm thick, yellowish-brown (4C5) to brown (5D7–5D8), fibrous to corky. Tube up to 0.1 cm long, brown (5C5), indistinctively stratified. Stipe up to 12.5 cm long and 4 mm diameter, cylindrical to subcylindrical, solid, fibrous to woody; surface brownish-black (8F7), glabrous to bumpy, laccate.

Hyphal system trimitic; generative hyphae with clamp connections; all hyphae IKI–, CB+; tissues darkening in KOH. Generative hyphae in context 1.2–2 µm diameter, colorless, thin-walled; skeletal hyphae in context 3.8–5 µm diameter, pale yellowish-brown, thick-walled with a wide to narrow lumen or sub-solid, arboriform and flexuous; binding hyphae in context 1.2–3.2 µm diameter, colorless, thick-walled, branched and flexuous. Generative hyphae in tubes 1–2 µm diameter, colorless, thin-walled; skeletal hyphae in tubes 4–6.5 µm diameter, colorless, thick-walled with a wide to narrow lumen or sub-solid, arboriform and flexuous; binding hyphae in tubes 1–3 µm diameter, colorless, thick-walled, branched and flexuous. Pileipellis composed of clamped generative hyphae, thick-walled; apical cells 25–45 × 7–14 μm, clavate, inflated, dark brown, anticlinal, forming a regular palisade. Stipitipellis composed of clamped generative hyphae, thick-walled; apical cells 20–40 × 5–10 μm, clavate, inflated, dark brown, anticlinal, forming a regular palisade. Cystidia and cystidioles absent. Basidioles 20–25 × 9–20 μm, subclavate to clavate, colorless, thin-walled. Basidia not observed. Basidiospores 10–11.5 × 7–8.5 μm, L = 10.44 μm, W = 7.55 μm, Q = 1.38 (n = 20/1/1, with myxosporium), 7.5–9.5 × 5–8 μm, L = 8.17 μm, W = 6.72 μm, Q = 1.12 (n = 20/1/1, without myxosporium), ellipsoid to broadly ellipsoid, pale yellowish-brown, IKI –, CB +, double-walled with slightly thick walls, exospore wall smooth, endospore wall with dense spinules. 

**Habitat:** Solitary or gregarious, occurring on decaying hardwood (often underground) in forests predominantly composed of fagaceous trees. 

**Known distribution:** Southern China (Hainan Province). 

**Notes:** *Ganoderma shennongii*, commonly referred to as “Lingzhi Wang” on Hainan Island in tropical China, was historically misidentified as *G. flexipes* [40]. However, *G. flexipes* can be distinguished by its larger pores and narrower basidiospores, measuring 8.5–12 × 5.5–7.5 μm (see above). In addition to *G. flexipes*, *G. shennongii* is morphologically and phylogenetically closely related to *G. subflexipes*. Nevertheless, *G. subflexipes* exhibits smaller pores and narrower basidiospores, measuring 8–11.5 × 5–7.5 μm (see below). Furthermore, *G. shennongii* shares morphological similarities with *G. bambusicola*; however, the latter is characterized by a more blackish pileus, narrower basidiospores (9.5–12.5 × 5–7 μm), and a specific habitat preference for growing on dead bamboo roots (see above). 

**Figure 10:**
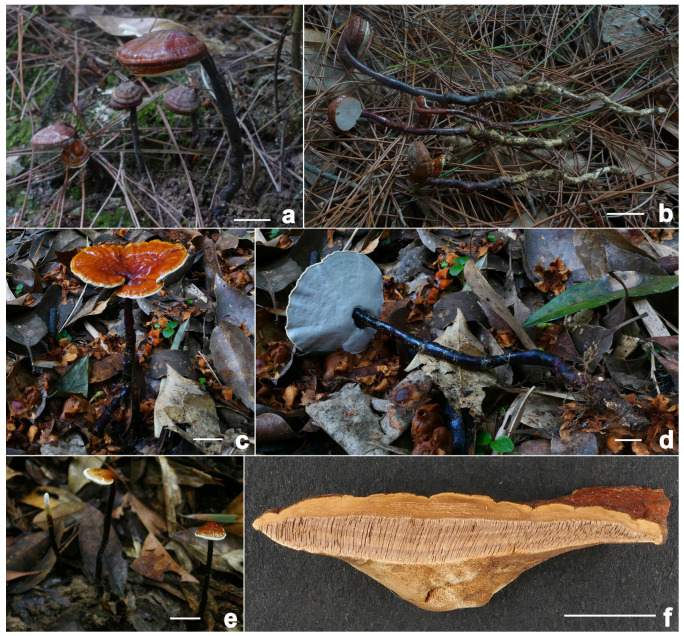
*Ganoderma subflexipes* (**a**–**e**) Basidiomata [(**a**,**b**) from FHMU2320; (**c**,**d**) from FHMU5725; (**e**) from FHMU2299]. (**f**) Section of pileus (FHMU5725). Scale bars = 1 cm. Photos by N.K. Zeng.

**Figure 11:**
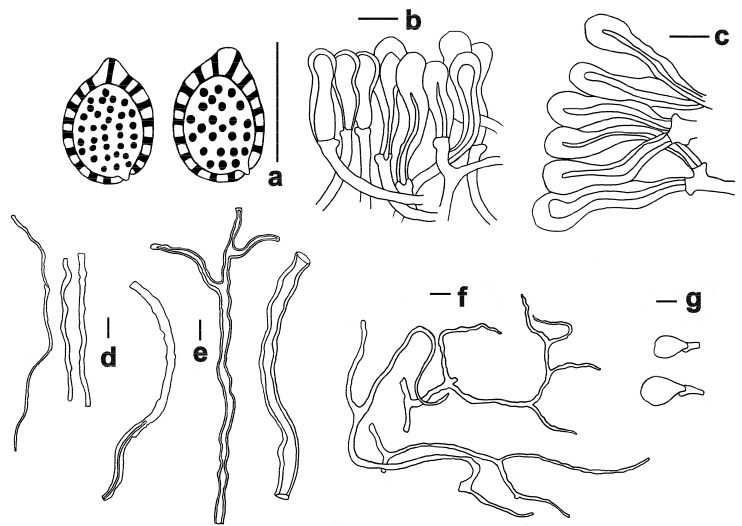
Microscopic features of *Ganoderma subflexipes* (FHMU2320). (**a**) Basidiospores. (**b**) Pileipellis. (**c**) Stipitipellis. (**d**) Generative hyphae. (**e**) Skeletal hyphae. (**f**) Binding hyphae. (**g**) Basidioles. Scale bars = 10 μm. Drawings by R. Tian.

***Ganoderma subflexipes*** B.K. Cui, J.H. Xing & Y.F. Sun, Stud. Mycol. 101: 350, 2022 (Figure 10 and Figure 11)

**MycoBank:** MB839675

**Description:** Basidiomata annual, eccentric or dorso-laterally stipitate, corky. Pilei up to 5 cm diameter and 0.5 cm thick, solitary, applanate, sub-reniform to reniform, or subflabellate to flabellate. Pileal surface reddish-brown (8D7–8D8) to orangish-brown (6B8), strongly laccate, glabrous when young, then bumpy, with conspicuously concentric furrows and slightly radial rugose; margin obtuse, entire, incurved. Pore surface white, turning yellowish-brown (4C5–4C6) when injured; pore 5–6 per mm, subcircular to circular or angular. Context up to 1.5 cm thick, yellowish-brown (4C5) to brown (5D7–5D8), fibrous to corky. Tubes up to 0.5 cm long, pale yellow (1A3–1A4) to grayish-yellow (4B4), indistinctively stratified. Stipe up to 15 cm long and 7 mm diameter, cylindrical to subcylindrical, solid, fibrous to woody; surface reddish-brown (7E7) to chocolate brown (8E7), glabrous to bumpy, laccate. 

Hyphal system trimitic; generative hyphae with clamp connections; all hyphae IKI–, CB+; tissues darkening in KOH. Generative hyphae in context 1–2 µm diameter, colorless, thin-walled; skeletal hyphae in context 2–6 µm in diameter, pale yellowish-brown, thick-walled with a wide to narrow lumen or sub-solid, arboriform and flexuous; binding hyphae in context 1–3.5 µm diameter, colorless, thick-walled, branched and flexuous. Generative hyphae in tubes 1.5–3 µm diameter, colorless, thin-walled; skeletal hyphae in tubes 3–5 µm in diameter, pale yellow, thick-walled with a wide to narrow lumen or sub-solid, arboriform and flexuous; binding hyphae in context 1–3.5 µm diameter, colorless, thick-walled, branched and flexuous. Pileipellis composed of clamped generative hyphae, thick-walled; apical cells 20–50 × 5–12 μm, clavate, inflated, dark brown, anticlinal, forming a regular palisade. Stipitipellis composed of clamped generative hyphae, thick-walled; apical cells 20–40 × 6–12 μm, clavate, inflated, dark brown, forming a regular palisade. Cystidia and cystidioles absent. Basidioles 15–19 × 8.5–13 μm, subclavate to clavate, colorless, thin-walled. Basidia not observed. Basidiospores 8–11.5 (–12) × 5–7.5 (–8) μm, L = 10.27 μm, W = 6.76 μm, Q = 1.52 (n = 120/6/3, with myxosporium), 6–9.5 × 4–7 μm, L = 7.78 μm, W = 5.34 μm, Q = 1.46 (n = 120/6/3, without myxosporium), ellipsoid to broadly ellipsoid, pale yellow, IKI–, CB+, double-walled with slightly thick walls, exospore wall smooth, endospore wall with dense spinules.

**Habitat:** It is solitary or gregarious and primarily found on dead roots of bamboo, particularly those of *Bambusa chungii* McClure. Occasionally, it may also occur on decaying hardwood (often underground) in forests dominated by fagaceous trees or in mixed forests where fagaceous trees and *Pinus massoniana* Lamb. are predominant.

**Known distribution:** Southern (Hainan and Guangdong Provinces), southeastern (Fujian Province), and eastern China (Jiangxi Province) [2].

**Specimens examined:** CHINA. Fujian Province: Zhangping City, Xingqiao Town, Chengkou Village, elev. 350 m, 20 August 2013, N.K. Zeng1455 (FHMU2320); Sanming City, Geshikao National Forest Park, elev. 380 m, 8 July 2013, Y.J. Hao969 (HKAS80249); Sanming City, Nature Reserve for Wild *Sarcandra glabra*, 8 July 2013, T. Guo724 (HKAS81926). Guangdong Province: Fengkai County, Heishiding Nature Reserve, elev. 360 m, 2 June 2013, Q. Cai924 (HKAS79603); Shaoguan City, Danxia National Nature Reserve, elev. 380 m, 5 June 2019, N.K. Zeng4086 (FHMU3731); same collection, 4 June 2019, N.K. Zeng4114 (FHMU5725). Hainan Province: Limushan of Hainan Tropical Rainforest National Park, elev. 650 m, 4 June 2009, N.K. Zeng242 (FHMU2299); Haikou City, 14 January 2015, bought from market, N.K. Zeng1893 (FHMU1218); same location and date, N.K. Zeng 1893-2 (FHMU7611). 

**Notes:** *Ganoderma subflexipes*, originally described in Guangdong in southern China [2], is commonly referred to as “Zhu Lingzhi” on Hainan Island in tropical China. This species is characterized by a reddish-brown to orangish-brown pileus, relatively small pores, a white pore surface, and a yellowish-brown to brown context. It typically grows on dead bamboo roots, though it is occasionally found on decaying hardwood (often underground).

## 4. Discussion

The species diversity of *Ganoderma* has been extensively documented in southern China, particularly on Hainan Island [2]. However, wild populations of *Ganoderma* species, including those referred to as “Lingzhi Wang” or “Zhu Lingzhi” on Hainan Island, have significantly declined due to overharvesting. Consequently, many species within this genus have been classified as priority protected species in the Hainan Tropical Rainforest National Park [76,77]. Effective conservation of these species necessitates accurate identification. Furthermore, there is an urgent need to reliably distinguish “Lingzhi Wang” or “Zhu Lingzhi”, which are crucial medicinal fungi in Hainan Island, from their close relatives, counterfeits, adulterants, and inferior substitutes to ensure their medicinal efficacy [78]. The present study identifies “Lingzhi Wang” as including *G. baisuzhenii*, *G. flexipes*, and *G. shennongii*, and “Zhu Lingzhi” as including *G. bambusicola* and *G. subflexipes*. The precise definition of these species provides essential data for the conservation and medicinal utilization of these *Ganoderma* species (Table 2).

In addition to the aforementioned species—namely, *G. baisuzhenii*, *G. bambusicola*, *G. flexipes*, *G. shennongii*, and *G. subflexipes*—other taxa such as *G. atrum* J.D. Zhao, L.W. Hsu & X.Q. Zhang, *G. calidophilum* J.D. Zhao, L.W. Hsu & X.Q. Zhang, *G. hainanense* J.D. Zhao, L.W. Hsu & X.Q. Zhang, *G. luteomarginatum* J.D. Zhao, L.W. Hsu & X.Q. Zhang, and *G. parviungulatum* J.D. Zhao & X.Q. Zhang, all originally described on Hainan Island in tropical China, are also characterized by their diminutive pilei and gracile stipes [79,80]. Cao et al. [11] proposed that *G. atrum*, *G. hainanense*, and *G. parviungulatum* are synonymous with *G. flexipes*, a suggestion later supported by Sun et al. [2]. Similarly, *G. luteomarginatum* was suggested to be synonymous with *G. sinense* J.D. Zhao, L.W. Hsu & X.Q. Zhang [2]. Although *G. calidophilum* was initially believed to be synonymous with *G. flexipes* [11,72], this viewpoint has been challenged in recent studies. The protologue of *G. calidophilum* does not align well with that of *G. flexipes*, as the former exhibits denser pores (4–6 per mm) and larger basidiospores, measuring 10–12.1 × 6.2–8.7 μm [79]. Consequently, the taxonomic relationship between *G. flexipes* and *G. calidophilum* remains unresolved and warrants further investigation in future studies.

**Figure 12:**
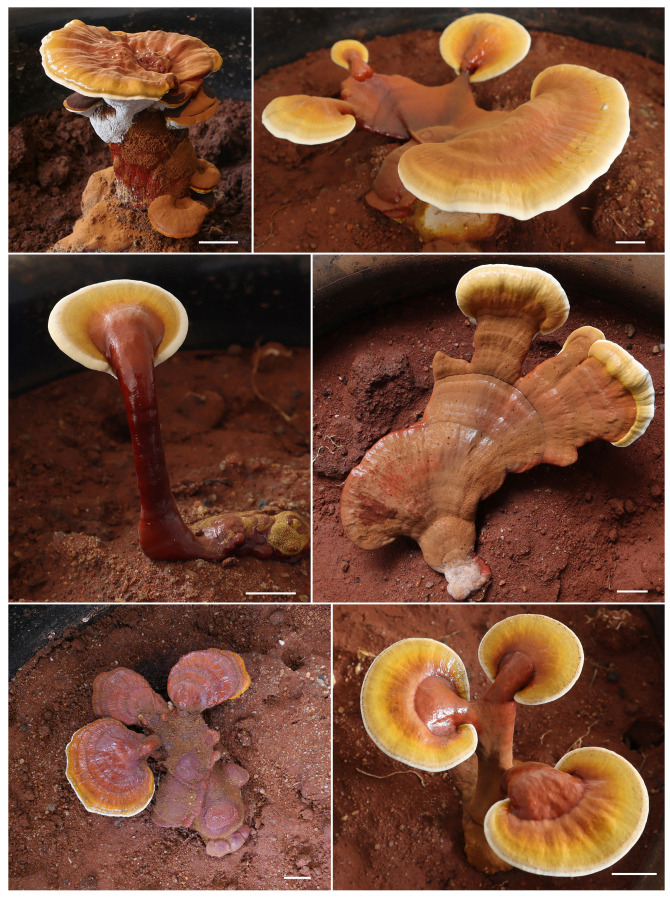
The varied fruit bodies from cultivated *G. subflexipes.* Scale bars = 1 cm. Photos by R. Tian.

Among *Ganoderma* species, high phenotypic plasticity at the macroscopic level is a well-documented phenomenon [81]. In a previous study [61], we successfully induced the formation of fruit bodies of *G. subflexipes* in a greenhouse environment. Notably, the cultivated *G. subflexipes* exhibited considerable morphological variation, with fruit bodies displaying diverse shapes (Figure 12). Additionally, the pilei of cultivated *G. subflexipes* were significantly larger, measuring up to 10 cm in length and 8.5 cm in width. These findings align with the observation that the morphological features of *G. subflexipes* fruit bodies are influenced by growing conditions, consistent with the views of Gilbertson and Ryvarden [82], who emphasized the high variability of fruit bodies in *Ganoderma* species. It is also noteworthy that the attachment type of the stipe to the pileus in *G. flexipes* exhibits considerable variation, ranging from lateral (Figure 6a–d) to nearly central (Figure 6e). This morphological variability underscores the challenges of relying solely on macroscopic characteristics for species identification. Therefore, in addition to morphological evaluation, DNA sequence data play a crucial role in the accurate identification of *Ganoderma* species. Integrating molecular data with traditional morphological approaches is essential for resolving taxonomic ambiguities and ensuring reliable species delineation.

DNA sequence data play a pivotal and indispensable role in resolving taxonomic delimitations within the genus *Ganoderma*, as evidenced by previous studies [1,2,3,40,51]. To conclusively address the taxonomic ambiguities surrounding “Lingzhi Wang” or “Zhu Lingzhi” specimens, comprehensive multilocus DNA phylogenetic analyses should be prioritized. Our current investigation has identified five candidate species corresponding to these vernacular names, yet this likely represents only a fraction of the taxonomic diversity. Notably, Hainan Island, a tropical biodiversity hotspot in China, harbors remarkable *Ganoderma* diversity [2,5]. We anticipate that future investigations incorporating advanced molecular techniques will reveal additional taxa characterized by the diagnostic morphological features of diminutive pilei and gracile stipes.

## 5. Conclusions

The present study reveals that the *Ganoderma* species characterized by small pilei and gracile stipes on Hainan Island in tropical China comprise at least five distinct species. Among these, two species—*G. baisuzhenii* and *G. shennongii*—are newly described. The remaining three species, *G. bambusicola*, *G. flexipes*, and *G. subflexipes*, have been previously documented. Notably, *G. bambusicola* is reported for the first time on the Chinese mainland. Furthermore, this study clarifies the taxonomic identities of “Lingzhi Wang” and “Zhu Lingzhi”, identifying “Lingzhi Wang” as encompassing *G. baisuzhenii*, *G. flexipes*, and *G. shennongii*, and “Zhu Lingzhi” as including *G. bambusicola* and *G. subflexipes*. These findings provide a foundation for the accurate identification, conservation, and sustainable utilization of these medicinally significant fungi.**Key to five *Ganoderma* species called “Lingzhi Wang” or “Zhu Lingzhi” in Hainan Island, tropical China**1. Host is bamboo21. Host is hardwood (especially Fagaceae trees)32. Pileus color is brownish-black, reddish-black to purplish-brown; basidiospore size 9.5–12.5 × 5–7 μm*G. bambusicola*2. Pileus color is reddish-brown to orangish-brown; basidiospore size 8–11.5 × 5–7.5 μm*G. subflexipes*3. Pileus context is nearly white; pore density up to 3 per mm; basidiospore up to 14 μm in length and 10 μm in width*G. baisuzhenii*3. Pileus context is brown, pore density up to 5 per mm; basidiospore up to 12 μm in length and 8.5 μm in width44. Pileus color is brownish-red to reddish; pore density 3–4 per mm; basidiospore size 8.5–12 × 5.5–7.5 μm *G. flexipes*4. Pileus color is dark reddish; pore density 4–5 per mm; basidiospore size 10–11.5 × 7–8.5 μm *G. shennongii*

## Figures and Tables

**Table 2:** Main characters of five *Ganoderma* species called “Lingzhi Wang” or “Zhu Lingzhi” in Hainan Island, tropical China.

Species	Local Name	Pilei Color	Pore	Context	Basidiospore (μm)	Host
*Ganoderma baisuzhenii*	Lingzhi Wang	Brownish-red to dark brownish-red	2–3 per mm; surface yellowish when young, then white	Nearly white	11.5–14 × 8–10	On decaying hardwood (often underground) of fagaceous trees, particularly those of the genus *Cyclobalanopsis*
*G. bambusicola*	Zhu Lingzhi	Brownish-black, reddish-black to purplish-brown	4–6 per mm; surface white	Dark brown	9.5–12.5 × 5–7	On dead roots of bamboo, particularly those of *Dendrocalamus latiflorus*
*G. flexipes*	Lingzhi Wang	Brownish-red to reddish	3–4 per mm; surface white	Upper layer yellowish-brown, lower layer brown to dark brown	8.5–12 × 5.5–7.5	On decaying hardwood (often underground) of fagaceous trees, particularly those of *Quercus patelliformis*
*G. shennongii*	Lingzhi Wang	Dark reddish	4–5 per mm; surface white	Yellowish-brown to brown	10–11.5 × 7–8.5	On decaying hardwood (often underground) in forests predominantly composed of fagaceous trees
*G. subflexipes*	Zhu Lingzhi	Reddish-brown to orangish-brown	5–6 per mm; surface white	Yellowish-brown to brown	8–11.5 × 5–7.5	On dead roots of bamboo, particularly those of *Bambusa chungii*, sometimes on decaying hardwood (often underground) in forests dominated by fagaceous trees, or mixed forests dominated by fagaceous trees and *Pinus massoniana*

## Data Availability

The datasets presented in this study have been deposited in NCBI GenBank (https://www.ncbi.nlm.nih.gov/genbank/ (accessed on 13 March 2025)) and Mycobank (https://www.mycobank.org/page/Home/MycoBank (accessed on 13 March 2025)).
